# The Shrinking Brain: Cerebral Atrophy Following Traumatic Brain Injury

**DOI:** 10.1007/s10439-018-02148-2

**Published:** 2018-10-17

**Authors:** Taylor C. Harris, Rijk de Rooij, Ellen Kuhl

**Affiliations:** Stanford University, Stanford, CA USA

**Keywords:** Traumatic brain injury, Cerebral atrophy, Neurodegeneration, Computational simulation, Finite element modeling

## Abstract

Cerebral atrophy in response to traumatic brain injury is a well-documented phenomenon in both primary investigations and review articles. Recent atrophy studies focus on exploring the region-specific patterns of cerebral atrophy; yet, there is no study that analyzes and synthesizes the emerging atrophy patterns in a single comprehensive review. Here we attempt to fill this gap in our current knowledge by integrating the current literature into a cohesive theory of preferential brain tissue loss and by identifying common risk factors for accelerated atrophy progression. Our review reveals that observations for mild traumatic brain injury remain inconclusive, whereas observations for moderate-to-severe traumatic brain injury converge towards robust patterns: brain tissue loss is on the order of 5% per year, and occurs in the form of generalized atrophy, across the entire brain, or focal atrophy, in specific brain regions. The most common regions of focal atrophy are the thalamus, hippocampus, and cerebellum in gray matter and the corpus callosum, corona radiata, and brainstem in white matter. We illustrate the differences of generalized and focal gray and white matter atrophy on emerging deformation and stress profiles across the whole brain using computational simulation. The characteristic features of our atrophy simulations—a widening of the cortical sulci, a gradual enlargement of the ventricles, and a pronounced cortical thinning—agree well with clinical observations. Understanding region-specific atrophy patterns in response to traumatic brain injury has significant implications in modeling, simulating, and predicting injury outcomes. Computational modeling of brain atrophy could open new strategies for physicians to make informed decisions for whom, how, and when to administer pharmaceutical treatment to manage the chronic loss of brain structure and function.

## Introduction

**Figure 1:**
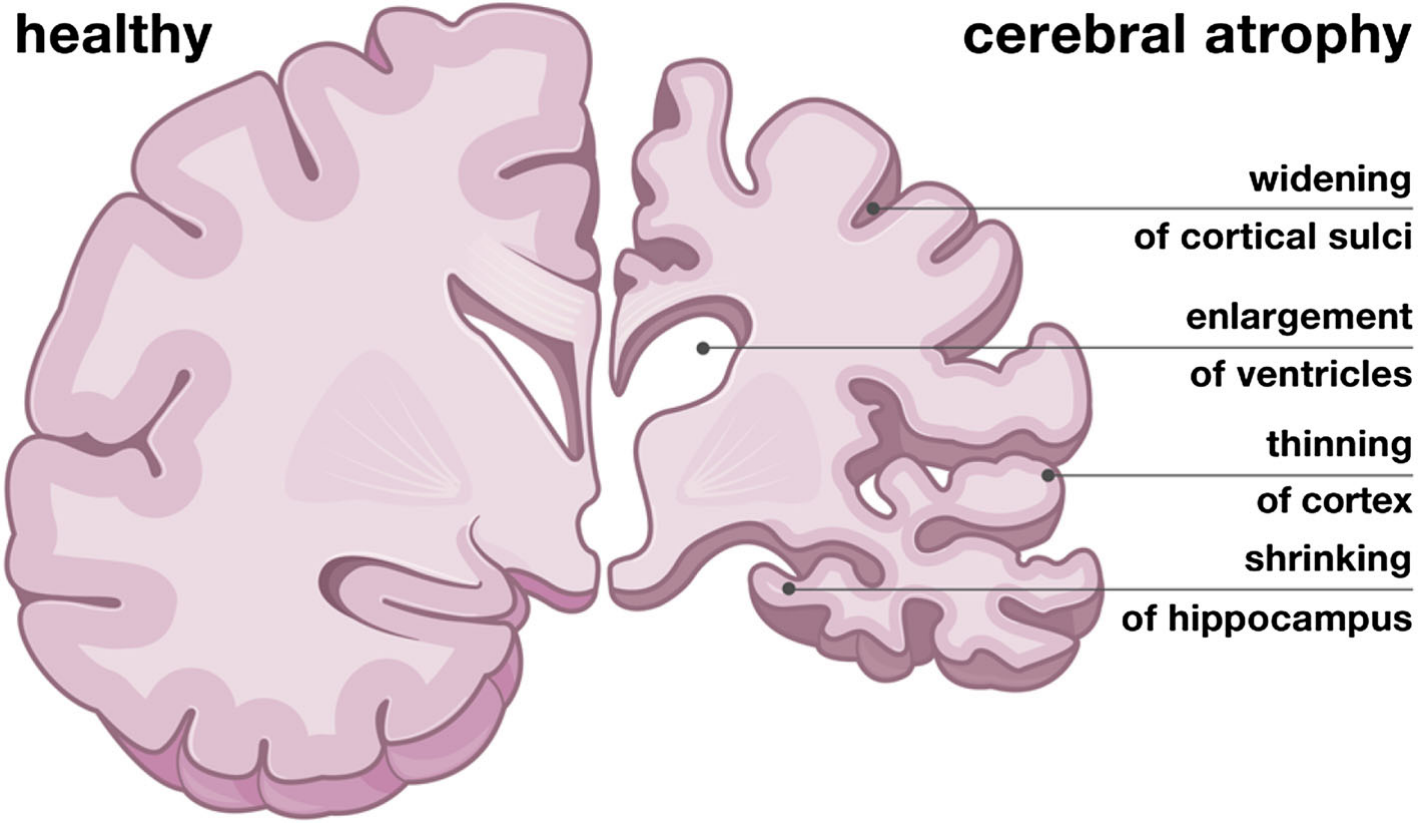
Characteristic features of cerebral atrophy following traumatic brain injury. Compared to the healthy brain, left, the brain in cerebral atrophy following traumatic brain injury experiences a widening of the cortical sulci, a gradual enlargement of the ventricles, a pronounced cortical thinning, and a shrinking of the hippocampus, right.

In 2013, emergency departments in the US recorded a total of 2.8 million visits related to traumatic brain injuries; 280,000 resulted in hospitalization and 56,000 in deaths.^56^ While these statistics capture the pervasive nature of traumatic brain injury, they fail to represent the $60 billion financial burden brain injury exerts on both the healthcare system and the economy at large given diminished worker productivity.^21^ Traumatic brain injury is the alteration of brain function caused by an external mechanical force to the head. The phase of primary injury, within the first milliseconds, is associated with an immediate biomechanical damage of the tissue in response to excessive stretch, compression, and shear.^14^ The phase of secondary injury, from minutes to days after the insult, involves complex biochemical cascade of events associated with inflammation, swelling, and an increase of the intracranial pressure.^64^ Long term, throughout months, years, or even decades, these events may result in structural and functional changes of the brain associated with cerebral atrophy, the gradual loss of neurons and the connections between them, and neurodegeneration, the gradual functional decline.^45^ Figure 1 highlights the characteristic features of cerebral atrophy following traumatic brain injury: a widening of the cortical sulci, a gradual enlargement of the ventricles, a pronounced cortical thinning, and a shrinking of the hippocampus.

**Figure 2:**
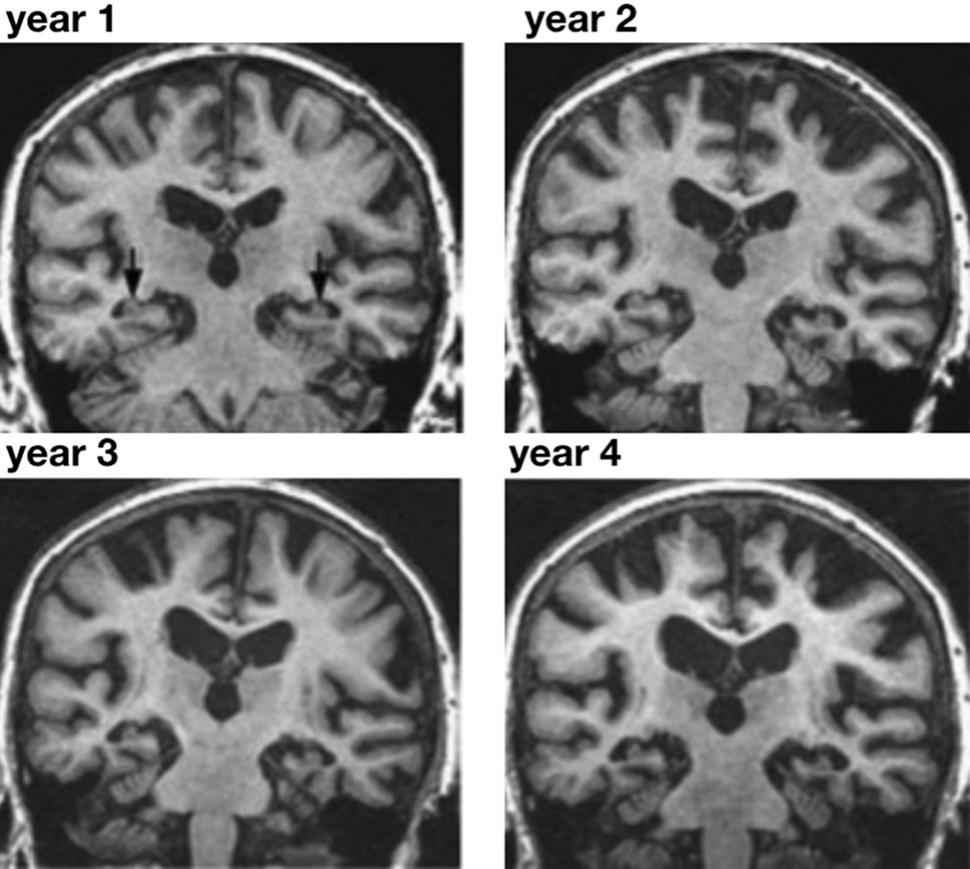
Cerebral atrophy in neurodegeneration. Longitudinal magnetic resonance imaging of an Alzheimer’s patient reveals the characteristic pattern of progressive atrophy in the hippocampus, a widening of the cortical sulci, a gradual enlargement of the ventricles, a pronounced cortical thinning, and a shrinking of the hippocampus. Adopted with permission from Ref. 39.

Traditionally, scientists have viewed the disease mechanisms of neurodegenerative disorders—the rapidly changing biomechanical environment during head impact and the slowly changing biochemical environment during neurodegeneration—as distinct and independent events. More recent studies are beginning to link neurodegenerative disease progression to mechanical risk factors.^46^ For example, it is increasingly recognized that chronic traumatic encephalopathy and Alzheimer’s disease share common degenerative pathways on the molecular and cellular levels, yet with a different pathological presentation^61^: neurofibrillary tangles of tau protein are present in both Alzheimer’s disease and chronic traumatic encephalopathy, but both emerge in distinct spatio-temporal patterns or stages; laminar amyloid-β plaques are present in Alzheimer’s disease but not in chronic traumatic encephalopathy; and TDP43 pathology is frequently observed in chronic traumatic encephalopathy but not in Alzheimer’s disease.^60^ Both chronic traumatic encephalopathy and Alzheimer’s disease manifest themselves through a symmetric atrophy of the frontal and temporal lobes, while the mammillary bodies and substantia nigra display marked atrophy in chronic traumatic encephalopathy but not in Alzheimer’s disease.^45^ Figure 2 illustrates the common characteristics of neurodegeneration and progressive cerebral atrophy^45^—a widening of the cortical sulci, a gradual enlargement of the ventricles, and a pronounced cortical thinning—by means of annual magnetic resonance images from the same Alzheimer’s patient.^39^ Indeed, athletes and military veterans with frequent exposure to moderate-to-severe head injuries are known to be at a greater risk of developing cerebral atrophy and dementia than the general population.^24^ Using machine learning on more than 1500 magnetic resonance images, a recent study observed an accelerated atrophy after traumatic brain injury and found that the brains of traumatic injury patients were on average 5 years older than their chronological age.^11^

While we increasingly recognize the role of physical forces in the advancement of neurodegeneration, functional and structural degradation develop gradually over years if not decades and their symptoms often remain undetectable until decades after the initial insult.^25^ The more immediate and directly assessable effects of closed-head impact result from strain and strain rate in the brain.^1^ In fact, the general consensus amongst experts is that lasting issues caused by traumatic brain injury result from elevated shear that generates diffuse axonal injury.^35^ The long-term degeneration catalyzed by shear is known as Wallerian degeneration,^62^ which, simply put, is axonal degeneration following injury that detaches the axon from the cell body.^52^ A second possibility of mechanically-induced structural failure of brain tissue is microtubule buckling along the axon at the time of impact. Structural damage to microtubules disrupts the intracellular transport, triggers neuronal inflammation, and causes axonal degeneration.^55^ Mechanical modeling and computational simulations can correlate microtubule polymerization and cross-link dynamics to axonal damage,^15^ characterize spatio-temporal patterns of stress, strain, and strain rates in response to mechanical loading,^28^ and help identify critical risk criteria on the whole brain level.^17^ In a cortical computational model,^26^ strain and strain rates in the brain during acute impact localized more in the sulci than in the gyri, which also corresponds to the spatial accumulation of tau proteins in deep sulcal regions in chronic traumatic encephalopathy pathology. In general, the greatest deformations occur ipsilateral and subjacent to the position of impact.^54^

Mechanical strain of brain tissue not only disturbs the structural components of the cell but it also triggers a cascade of molecular responses.^29^ The immediate molecular effect of strain on neurons is an ion imbalance with an increase in extracellular potassium and intracellular calcium levels. Hypermetabolism follows, as measured by glucose metabolism, for up to 3 h; this period of activity gives way to hypometabolism onset, which can last up to 5 days.^69^ Increased glutamate and aspartate release from neurons at time of impact correlates positively with injury severity and contributed to metabolic depression.^19^ Glutamate release has been further associated with blood–brain barrier compromise as part of a molecular cascade that leads to further inflammation, reduced oxygen perfusion, and, finally, degeneration.^54^ Thus, primary brain volume decline occurs within the first 6 months after injury with an approximately 10% volume decrease, the equivalent of decades of aging.^4^

The objective of this review is to more clearly elucidate the spatial patterns of brain atrophy following traumatic brain injury. Understanding the detailed nature of both the extent of atrophy and the regions most significantly affected can provide clinical insight into long-term predictions for neuropsychiatric outcomes. Similarly, such information can guide the development of computational models for predicting the outcome of traumatic brain injury-induced atrophy. Mechanical factors, such as strain and stress, that evolve during the course of tissue atrophy may prove important in stimulating further disease progression, but have yet to be probed. Research into the mechanics of neurodegeneration as a chronic factor in atrophy may elucidate its role as a propagating agent in tissue atrophy.

## Other Mechanisms of Atrophy

Cerebral atrophy is a well-documented pathological outcome that is shared by a magnitude of neurodegenerative conditions. While our review focuses on biomechanically-induced volumetric decline, we note that atrophy could also be a result of biochemically-induced phenomena associated with disease or aging.

### Disease

A number of diseases that impact the brain also simultaneously generate atrophy including Alzheimer’s disease,^5^ Parkinson’s disease,^8^ Huntington’s disease,^36^ multiple sclerosis,^42^ and even infectious diseases like AIDS.^13^ Recent studies have shown that chronic traumatic encephalopathy also causes atrophy. Although it is unclear whether closed-head traumatic brain injuries directly correlate with likelihood of developing Alzheimer’s disease,^24^,^48^ there is growing evidence for a causal relationship between repetitive clinical and subclinical traumatic brain injuries and chronic traumatic encephalopathy.^9^,^24^,^26^ Regardless, the clinical outcomes of a mechanically induced dementia, chronic traumatic encephalopathy, and arguably more biological ones, stroke and Alzheimer’s disease, are very similar; in fact, both result in tau protein aggregation, brain atrophy, and deterioration of memory.^4^

### Aging

Brain volume decreases as part of the natural aging process. The process of pruning in the gray matter tissue begins as early as childhood, whereas white matter reduction begins midlife. At around age 35, the overall brain volume begins decreasing at an approximate rate of − 0.2% per year, a rate that decreases to − 0.5% per year at age 60.^4^ This review will show that the magnitude of atrophy caused in 1 year by closed-head injuries equates to several years of natural aging; in fact, a study reported that the brain volume of moderate-to-severe 52-year-old traumatic brain injury survivors matched 71-year-old individuals with dementia.^49^

## Methods

### Literature Inclusion Criteria

Brain volume atrophy after traumatic brain injury has been well documented in the literature. There is widespread acceptance amongst researchers that brain volume decreases significantly following closed-head traumatic brain injury events. However, documentation of spatial patterns of atrophy is less ubiquitous. As such, a thorough review of the available literature is critical to synthesize the dispersed information regarding patterns of atrophy and the correlating clinical parameters. In this review, we only include investigations of atrophy patterns in which participants have been clearly segmented into mild, moderate, and severe injury survivors. Investigations that include traumatic brain injury participants of all injury severities risk confounding the results depending on the proportions of degree of injury; we will discuss this further when we address limitations in the current literature. Furthermore, we exclude any studies that focus on pediatric patients. Given that pediatric patients are still in a phase of growth, any results of brain atrophy in the pediatric population will be impacted by natural growth progressions. As such, we restrict our review to the adult population in an attempt to reduce factors that may skew our conclusions and interpretation of the data in the literature.

### Magnetic Resonance Imaging

All studies we included in this review used T1-weighted magnetic resonance images for the analyses. In T1 images, black equates to gaseous material and areas of high mineral density or blood flow, dark gray to areas with high water content, light gray to areas with high protein content, and white to fat.^22^ T2 images, in which some types of tissue may show up in two different color regimes, were also collected in some studies but were not used in any of the analyses. The fact that T1 images cleanly delineate structures within the image explains the bias within the literature to use T1-weighted magnetic resonance imaging over T2 imaging. However, T2 images could become useful in verifying the degree of brain tissue swelling.

### Voxel-Based Morphometry

Except for one outlier, all studies of this review analyzed magnetic resonance images using either voxel- or surface-based morphometry. Various software packages can perform automated voxel-based morphometry with magnetic resonance images as input. To normalize structural differences in three dimensions, the general workflow requires an initial mapping of the patient brain to a generic brain atlas.^2^ After normalization, the images are segmented into the three main types of material in the brain, gray matter, white matter, and cerebrospinal fluid. Finally, the software calculates concentrations of each material type within each voxel and performs statistical analyses to determine structural differences between populations.^2^

### Surface-Based Morphometry

In surface-based morphometry, the primary goal is to extract geometric models of key features of the human brain from magnetic resonance images. The main and first feature to extract is the cortical surface of the brain, the outer brain layer. Then, surface-based morphology extracts the surfaces between white and gray matter and between gray matter and cerebral spinal fluid. From the resulting geometric models, we can extract cortical thicknesses and gray and white matter tissue volumes. The open source software package FreeSurfer is the most popular tool for surface based morphometry.^12^

### Tensor-Based Morphometry

Only one study used tensor-based morphometry to analyze the magnetic resonance images.^52^ Similar to voxel-based morphometry, tensor-based morphometry also requires a template brain, but instead of using a generic atlas, the template is an aggregated average of all control images to be included in analysis.^33^ An algorithm alters the subject-specific magnetic resonance images to match the template and determines the deformation **φ** necessary to align subject images with controls. From the deformation **φ**, the authors calculated the deformation gradient, **F** = **∇φ**, and the Jacobian *J* = det(**F**), which characterizes the volume changes in the study population.^33^

## Regional Atrophy Patterns in Mild Injury

Cerebral atrophy can affect different parts of the brain. Generalized atrophy affects cells across the entire brain whereas focal atrophy affects specific brain regions and results in a loss of function associated with those areas. Very few investigations in the current body of literature focus solely on brain atrophy in individuals who have sustained mild injuries. As a result, our review of brain atrophy outcomes for this population is substantially smaller than the review for moderate-to-severe individuals. Table 1 summarizes the effects of mild traumatic brain injury on whole brain, gray matter, and white matter atrophy.

**Table 1:** Cerebral atrophy in mild traumatic brain injury. Effects on the whole brain, gray matter tissue, and white matter tissue.

	Brain regions	Volume loss	Study types	Number of subjects	References
Whole brain	Parenchyma	− 4.16%	Longitudinal: 350 days	Injured: 14 Control: 10	MacKenzie *et al*.^43^
Gray matter	Left pericalcarine	–	Cross-sectional	Injured: 8 Control: 25	Spitz *et al*.^53^
White matter	–	–	–	–	–

### Whole Brain

A comprehensive longitudinal study compared the change in percent brain parenchymal volume (*%VBP* = [parenchymal volume]/[parenchymal volume + cerebral spinal fluid]) over time between TBI subjects and controls.^43^ Of the 14 traumatic brain injury individuals, 11 had sustained mild injury as determined by the Mild Traumatic Brain Injury Interdisciplinary Special Interest Group of the American Congress of Rehabilitation Medicine. The group obtained magnetic resonance images of participants at two time points, approximately 350 days apart. The first image was recorded an average of 125 days post injury. When compared to controls the traumatic brain injury group displayed a significantly greater change in *%VBP* at an average of − 4.16% compared to − 1.49% in the controls.

### Gray Matter

A study of mild traumatic brain injury patients 20 months post injury revealed a pronounced decrease in gray matter volume.^53^ Specifically, the pericalcarine region exhibited the greatest volume decrease. The study included eight participants classified as having mild traumatic brain injury. Interestingly, the study used the duration of post-traumatic amnesia as a proxy for classifying injury severity, which deviates from the general consensus of using the Glasgow Coma Scale and a few other parameters to determine severity. Subjects with post-traumatic amnesia lasting less than 24 h were classified to have mild injury. Finally, unlike the aforementioned study on mild injury,^43^ this investigation was not longitudinal but rather cross-sectional. Magnetic resonance images of traumatic brain injury patients were obtained approximately 20 months after the injury event. Regional patterns of atrophy emerged when comparing traumatic brain injury-associated magnetic resonance images with those from a control group, which was matched for age, gender, and education. Hence, there are no quantitative results in terms of atrophy extent.

### White Matter

Of studies focusing only on the mild traumatic brain injury subpopulation, none reported specific changes in the white matter.

## Regional Atrophy Patterns in Moderate-to-Severe Injury

The vast majority of regional atrophy studies currently available that control for injury severity focus on the moderate-to-severe injury subgroup.

### Whole Brain

A recent investigation, which included 61 participants with moderate-to-severe injury as determined by the Mayo Classification System, revealed quantitative differences in total brain volume during the chronic phase of traumatic brain injury.^10^ The longitudinal study acquired magnetic resonance images at two time points, at an average of 1 year after injury and at 1 year subsequent to the first. It is important to note that there was a wide range in both, the time since injury and the time between the two imaging time points. The group reported a change in − 1.51% total brain volume in this time period, which was significantly greater than the change in overall brain volume of the control group. While this investigation demonstrated the lasting impact of traumatic brain injury on brain atrophy throughout the chronic phase of injury, other investigations have quantified the overall volume loss beginning at the acute stage of injury. One study reported a − 8.5% overall brain tissue loss between the acute phase, 4–19 days after injury, and chronic phase, approximately 6 months after injury, using longitudinal magnetic resonance imaging of severe traumatic brain injury individuals.^44^ Similarly, another study acquired brain magnetic resonance images from severe traumatic brain injury patients at 8 weeks post injury and then 1 year post injury.^52^ The group reported a − 4% volume change in the traumatic brain injury group, which was significantly larger than the − 0.18% decrease seen in the control group. A third study measured a similar magnitude of volume decrease in brain parenchyma when following moderate-to-severe traumatic brain injury survivors with magnetic resonance imaging at a median of 1 day and then 8 months after injury.^63^ Specifically, the group found a − 4.5% volume change in brain parenchyma tissue. A few other investigations have also reported total brain volume atrophy *via* cross-sectional studies. A recent study found that total cortical volume in moderate-to-severe traumatic brain injury patients was significantly smaller than that measured in a control group.^27^ Another longitudinal study reported that moderate-to-severe traumatic brain injury patients exhibit a 1.32% increase in ventricle-to-brain ratio per month following the injury event, as compared to only 0.18% rate of ventricle-to-brain ratio increase per month in the control group.^30^ Though not a direct measure of total brain volume, the ventricle-to-brain ratio is commonly probed as a proxy for total brain atrophy in traumatic brain injury patients. In this investigation, imaging was performed on average 5 and 20 months post injury; yet, similar to other studies previously mentioned, the time interval between injury and imaging varied widely. Both longitudinal and cross-sectional studies provide robust support, both quantitatively and qualitatively, for total brain atrophy in patients with sustained traumatic brain injury. Table 2 summarizes the effects of moderate-to-severe traumatic brain injury on the whole brain.

**Table 2:** Cerebral atrophy in moderate-to-severe traumatic brain injury. Effects on the whole brain.

Brain regions	Volume loss	Study types	Number of subjects	References
Global	− 1.51%	Longitudinal: 1 year	Injured: 61 Control: 32	Cole *et al*.^10^
	− 8.5%	Longitudinal: 6 months	Injured: 15 Control: 0	Marcoux *et al*.^44^
	− 4.5%	Longitudinal: 8 months	Injured: 25 Control: 22	Warner *et al*.^63^
	− 4%	Longitudinal: 10 months	Injured: 24 Control: 14	Sidaros *et al*.^52^
	–	Cross-sectional	Injured: 22 Control: 27	Gooijers *et al*.^27^
Ventricle-to-brain ratio	+1.32% per month	Longitudinal: 20 months	Injured: 56 Control: 12	Green *et al*.^30^

### Gray Matter

Of investigations with conclusions of gray matter atrophy, few report quantitative results. In a broad sense, gray matter volume decreases in the moderate-to-severe traumatic brain injury population in both overall volume of cortical and subcortical gray matter^27^ as well as in gray matter thickness.^47^,^59^ Specific patterns of gray matter atrophy occur in a wide range of structures. For example, in severe traumatic brain injury participants with a Glasgow Coma Score between 4 and 8 and with a median of 40 days post traumatic amnesia, significant differences emerged between the traumatic brain injury group and the control group in volume of the right parahippocampal gyrus, right putamen, upper vermis, and bilateral dorsomedial thalami.^50^ Similarly, a longitudinal study of moderate-to-severe traumatic brain injury participants 2 and 12 months post injury revealed significant atrophy in the bilateral thalamus and pallidum.^3^ Atrophy in the structures described above has repeatedly been confirmed by other studies. Specifically, the most commonly reported areas of gray matter atrophy were the bilateral thalamus,^3^,^10^,^27^,^40^,^50^^–^52 the bilateral hippocampus,^10^,^47^,^51^ the cerebellum,^10^,^51^ the bilateral putamen,^27^,^40^,^52^ the bilateral pallidum,^3^,^27^ and the insula.^10^,^51^ In addition, several disparate locations of gray matter atrophy have been documented including the left caudate^40^; the basal forebrain and dorsal tegmental^51^; the cuneus, the right superior frontal lobe, and the rostral middle frontal lobe.^53^ To our knowledge these areas have only been recognized once in the literature, so further research validating and replicating such outcomes is essential to confirming the significance of these regional volume declines.

In terms of quantitative differences in gray matter volume, we identified four studies, three cross-sectional and one longitudinal, with numerical estimates of specific atrophy. The most recent investigation found significant changes in the gray matter volume of the bilateral frontal, temporal, and occipital cortices.^10^ Specific regions that contributed significantly to the observed atrophy were the insula cortex, cerebellum, thalamus, hippocampus, and amygdala. Interestingly, the group determined that sulci atrophy more than gyri. Overall, there was a − 1.55% atrophy of gray matter during the chronic injury phase. While this investigation did not separately report volumetric changes in gray matter by the various structures that atrophied, other studies actually did. Cross-sectional analyses revealed that the hippocampal volume of 1.1 cm^3^ was − 27% smaller in moderate-to-severe patients in the chronic injury stage than in uninjured brains where it measured 1.512 cm^3^.^57^ A similar cross-sectional study documented cortical thinning by − 19% in the frontal lobe, − 11% in the temporal lobe, and − 15% in the occipital lobe for traumatic brain injury participants compared to controls. Finally, a longitudinal investigation monitored atrophy between the acute and chronic disease stages 1 day and 8 months post injury.^63^ The hippocampus decreased by − 10%, the thalamus by − 11%, and the amygdala by − 15%.

Looking at the qualitative and quantitative studies as a whole, we conclude that the most commonly reported areas of gray matter atrophy are in the thalamus, hippocampus, cerebellum, putamen, pallidum, insula, and amygdala. Furthermore, significant cortical thinning has been observed. Table 3 summarizes the effects of moderate-to-severe traumatic brain injury on gray matter atrophy.

**Table 3:** Cerebral atrophy in moderate-to-severe traumatic brain injury. Effects on gray matter tissue.

Brain regions	Volume loss	Study types	Number of subjects	References
Global	− 1.55%	Cross-sectional	Injured: 22 Control: 27	Gooijers *et al*.^27^
Cortical thickness	From − 10.7 to − 20.4%	Cross-sectional	Injured: 1 Control: 43	Turken *et al*.^59^
	− 0.11 mm	Cross-sectional	Insured: 26 Control: 22	Palacios *et al*.^47^
Bilateral thalamus	–	Longitudinal: 1 year	Injured: 61 Control: 32	Cole *et al*.^10^
	–	Longitudinal: 11 months	Injured: 35 Control: 36	Bendlin *et al*.^3^
	–	Longitudinal: 10 months	Injured: 26 Control: 14	Sidaros *et al*.^52^
	Varied by hemisphere	Cross-sectional	Injured: 22 Control: 27	Gooijers *et al*.^27^
	Varied by hemisphere	Cross-sectional	Injured: 20 Control: 26	Leunissen *et al*.^40^
	–	Cross-sectional	Injured: 22 Control: 23	Salmond *et al*.^51^
	–	Cross-sectional	Injured: 8 Control: 17	Ruet *et al*.^50^
Bilateral hippocampus	–	Longitudinal: 1 year	Injured: 61 Control: 32	Cole *et al*.^10^
	–	Longitudinal: 10 months	Injured: 26 Control: 14	Sidaros *et al*.^52^
	− 10%	Longitudinal: 8 months	Injured: 25 Control: 22	Warner *et al*.^63^
	–	Cross-sectional	Injured: 26 Control: 22	Palacios *et al*.^47^
	− 3.62 mm^3^	Cross-sectional	Injured: 22 Control: 23	Salmond *et al*.^51^
	− 0.412 cm^3^	Cross-sectional	Injured: 19 Control: 19	Tomaiuolo *et al*.^57^
	Varied by hemisphere	Cross-sectional	Injured: 22 Control: 27	Gooijers *et al*.^27^
	Varied by hemisphere	Cross-sectional	Injured: 20 Control: 26	Leunissen *et al*.^40^
Bilateral pallidum	–	Longitudinal: 11 months	Injured: 35 Control: 36	Bendlin *et al*.^3^
	Varied by hemisphere	Cross-sectional	Injured: 22 Control: 27	Gooijers *et al*.^27^
Cerebellum	–	Longitudinal: 1 year	Injured: 61 Control: 32	Cole *et al*.^10^
	–	Cross-sectional	Injured: 22 Control: 23	Salmond *et al*.^51^
Insula	–	Longitudinal: 1 year	Injured: 61 Control: 32	Cole *et al*.^10^
	–	Cross-sectional	Injured: 22 Control: 23	Salmond *et al*.^51^

### White Matter

The most prevalent hypothesis in the field regarding the mode through which lasting traumatic brain injury-induced degradation of white matter occurs is Wallerian degradation.^62^ Wallerian degeneration is non-specific to injury modality, and it generally proceeds as a result of nerve damage in which the axon is sheared from the cell body. The disconnected axons degrade and produce a net tissue volume decrease.^52^ Similar to our discussion on gray matter atrophy, we begin with qualitative interpretations of white matter volume change and then move to quantitative ones.

A number of white matter structures have been reported in the literature to display significant volumetric decreases following traumatic brain injury in the moderate-to-severe population; some have only been reported once while others have been observed by multiple disparate sources. The six most commonly identified white structures with significant qualitative atrophy are the corpus callosum,^3^,^10^,^52^,^58^ the corona radiate,^3^,^10^,^52^ the capsule,^3^,^10^,^51^,^52^,^58^ the brainstem,^10^,^52^ and the inferior and superior fascicules.^3^,^50^ Various other structures have also been reported individually in the literature. For example, the periventricular area decreased significantly,^50^ which supports previously mentioned findings of ventricle-to-brain ratio increase after traumatic brain injury.^30^ More specific areas include the cingulum, parts of the cerebellar peduncles, corticospinal tract^3^; the forceps major, fornix, and the chiasma.^58^

Quantitatively, white matter was reported to shrink − 5.8% in the first 8 months following injury.^63^ Then, in the chronic interval between 1 and 2 years after injury, the white matter continued to shrink by − 1.49%.^10^ In the first 6 months post-injury, however, one study observed a − 12% volume decrease in the white matter of the frontal lobe^44^; another study found a − 7.5% volume decrease in the white matter of the temporal lobe.^68^ Although the differences between contralateral and ipsilateral sides were not reported, the lobes closer to regions of the hemorrhage atrophied almost two times as much as the lobes away from the hemorrhage. On an even more granular level, several investigations described significant volume losses and even shape changes of specific structures. The corpus callosum in traumatic brain injury patients with 0.699 cm^3^ was − 14% smaller than in the control group with 0.814 cm^3^ and was also more concave.^58^ Longitudinal results support these cross-sectional results with an observed decrease of − 21.4% in the middle anterior corpus callosum, − 13.6% in the central corpus callosum, and − 16.8% in the middle posterior corpus callosum.^63^ The first study also observed a decrease in the fornix from 0.738 to 0.633 cm^3^ but with preserving the original shape,^58^ while the second study did not report volume changes in the fornix, but rather in the brain stem by − 6%.^63^ Table 4 summarizes the effects of moderate-to-severe traumatic brain injury on white matter atrophy.

**Table 4:** Cerebral atrophy in moderate-to-severe traumatic brain injury. Effects on white matter tissue.

Brain regions	Volume loss	Study types	Number of subjects	References
Global	− 5.8%	Longitudinal: 8 months	Injured: 25 Control: 22	Warner *et al*.^63^
	− 1.49%	Longitudinal: 1 year	Injured: 61 Control: 32	Cole *et al*.^10^
Corpus callosum	–	Longitudinal: 11 months	Injured: 35 Control: 36	Bendlin *et al*.^3^
	–	Longitudinal: 1 year	Insured: 61 Control: 32	Cole *et al*.^10^
	–	Longitudinal: 10 months	Injured: 24 Control: 14	Sidaros *et al*.^52^
	− 0.115 cm^3^	Cross-sectional	Insured: 19 Control: 19	Tomaiuolo *et al*.^58^
Corona radiata	–	Longitudinal: 11 months	Injured: 35 Control: 36	Bendlin *et al*.^3^
	–	Longitudinal: 1 year	Insured: 61 Control: 32	Cole *et al*.^10^
	–	Longitudinal: 10 months	Injured: 24 Control: 14	Sidaros *et al*.^52^
Capsule	–	Longitudinal: 11 months	Injured: 35 Control: 36	Bendlin *et al*.^3^
	–	Longitudinal: 1 year	Insured: 61 Control: 32	Cole *et al*.^10^
	–	Cross-sectional	Injured: 22 Control: 23	Salmond *et al*.^51^
	–	Longitudinal: 10 months	Injured: 24 Control: 14	Sidaros *et al*.^52^
	–	Cross-sectional	Injured: 19 Control: 19	Tomaiuolo *et al*.^58^
Inferior and superior fascicules	–	Cross-sectional	Injured: 8 Control: 17	Ruet *et al*.^50^
	–	Longitudinal: 11 months	Injured: 35 Control: 36	Bendlin *et al*.^3^
Brainstem	–	Longitudinal: 1 year	Injured: 61 Control: 32	Cole *et al*.^10^
	–	Longitudinal: 10 months	Injured: 24 Control: 14	Sidaros *et al*.^52^
	–	Longitudinal: 8 months	Injured: 25 Control: 22	Warner *et al*.^63^

## Correlative Factors

Recent investigations have not only investigated the spatial patterns of tissue degradation in response to mechanical injury but also drawn conclusions regarding correlations between clinical parameters and the magnitude of atrophy.

### Injury Severity

All studies in the current literature quantified injury either fully or partially using the patient’s lowest Glasgow Coma Score within 24 h of injury. Some considered loss of consciousness duration, post-traumatic amnesia, and focal lesions to revise conclusions drawn by the Glasgow Coma Score alone. These clinical parameters are routinely recorded when determining the severity of traumatic brain injury, which make them especially attractive as possible predictive markers of the degree of brain atrophy. The Glasgow Coma Score correlated with atrophy in the white matter, specifically in the fornix, cerebellar peduncles, cingulum, corona radiata, and inferior longitudinal fasciculus, and in the gray matter, specifically in the caudate and superior parietal regions.^3^ Yet, these conclusions are dubious at the moment, as another investigation of mild traumatic brain injury subjects found that the Glasgow Coma Score was not correlated with extent of atrophy.^43^ When comparing differences between the mild and moderate-to-severe groups, however, severity in the acute phase of injury, as determined by the Glasgow Coma Score, loss of consciousness, post-traumatic amnesia, and focal lesions, was correlated to greater volume loss.^41^ Similar to the conflicting conclusions regarding severity metrics and tissue loss in mild traumatic brain injury, investigations of moderate-to-severe injury also report opposing views. For example, one study found a correlation of the Glasgow Coma Score and post-injury epilepsy with tissue loss in cortical regions including the transverse frontopolar gyri, middle frontal gyri, lingual gyri, and angular gyri, while other areas of atrophy were supposedly correlated with the Glasgow Coma Score alone.^34^ Another study did not observe a correlation between the Glasgow Coma Score and atrophy in a study of severe traumatic brain injury survivors.^68^ Interestingly, the duration of loss of consciousness correlated with a specific atrophy in the corpus callosum by − 3.863 cm^3^ lost per day of coma in patients with severe traumatic brain injury.^57^

### Age

Few studies directly probe a link between age and volume loss following traumatic brain injury. A recent study determined a positive correlation between age and atrophy magnitude and observed an extensive effect with greatest significance in the cortical regions of the temporal and parietal lobes.^53^ Age also may correlate with the decline of some white matter structures, specifically the fornix.^57^

### Molecular Markers

The use of catheters in monitoring the composition of the cerebrospinal fluid following traumatic brain injury has provided insight into possible correlations between metabolic byproducts and tissue degradation. Lactate–pyruvate ratios greater than 40 in the first 96 h after moderate-to-severe injury predicted increased volume loss after 6 months.^44^ While this study did not observe correlations between glucose metabolism or glutamate levels and frontal lobe tissue loss, another investigation did observe a relationship between glucose metabolism and frontal lobe atrophy.^68^ As with age, the field is sparse with literature on the predictive ability of molecular markers, but unlike age, the conclusions regarding molecular markers are contradictory.

## Current Limitations in the Literature

In the process of reviewing the literature, we noticed several limitations in individual studies that translate to trends across the field. The most obvious is that almost all studies had very small sample sizes. One cross-sectional study even used an experimental group of a single person with a severe injury.^59^ Although this was an outlier, all but two studies had fewer than *n* = 25 traumatic brain injury subjects; one study had *n* = 35^3^ and one had *n* = 61.^10^ Along with small sample sizes, the vast majority of participants were male. Not only do small sample sizes implicate the statistical significance, but the low proportions of women make it hard to conclude whether observations in the literature translate to the female population.

A further limitation that seems to pervade the experimental landscape is the regulation of time between injury and imaging collection. Although some studies specifically controlled for time when assessing acute and chronic injury, many imaged the subjects at heterogeneous time points. In some studies, magnetic resonance images were collected between 3 and 113 months following injury.^57^ Such experimental designs conflate acute and chronic injury, which obscures information regarding atrophy rate.

When considering all studies as a body of work upon which to synthesize individual conclusions into a cohesive theory, we remain somewhat skeptical about the strength of some conclusions. This is, at least in part, due to deviations in experimental design amongst some investigations. For example, some studies chose to exclude patients with large focal lesions^43^,^63^ whereas others included patients with focal lesions without masking them in volumetric analyses.^51^,^53^ Such experimental decisions may influence quantitative outcomes differently and impact our ability to broad conclusions.

Similarly, the use of various software modalities through which to perform volumetric analyses, as discussed above, may also contribute to different conclusions regarding significant patterns of atrophy. Many but not all investigations used FreeSurfer,^12^ yet the brain atlases FreeSurfer employs are calibrated to healthy brains and the software falters when handling the blurry line between gray and white matter induced by shear injuries.^53^ To correct for such insufficiencies, many investigations manually redefined boundaries around areas of interest, which is also susceptible to human error.

Finally, the field taken as one entity lacks rigorous investigations regarding mild traumatic brain injury. The vast majority of the studies reviewed here focused on the moderate-to-severe population, which has rendered concluding on spatial atrophy in mild traumatic brain injury subjects somewhat nebulous. Beyond understanding atrophy after an isolated mild traumatic brain injury, investigating the mild traumatic brain injury population, especially with a view towards repeated concussive or sub-concussive injuries, may also provide insights into chronic traumatic encephalopathy disease progression.^45^ To date, it appears that the literature on chronic traumatic encephalopathy is dominated by post-mortem studies that report significant neurodegeneration,^24^ but investigations on the mechanisms of disease progression and rigorous testing of spatial atrophy have yet to be performed. Promising studies are underway to correlate observed injury patterns to the to the magnitude and location of the impact *via* finite element analysis,^20^ but more sophisticated analyses are needed to calibrate and validate finite element-based technologies as a reliable platform for injury prediction.^28^

While we did encounter several studies that enrolled mild traumatic brain injury participants,^18^,^23^,^30^,^38^ we decided to not include these studies because they did not analyze the mild and moderate-to-severe subjects separately. With increasing evidence supporting the correlation between injury severity and extent of atrophy, any new investigation should classify study subjects by injury severity. We suspect that observations of significant volume change in studies that span both groups are biased by a comparatively greater atrophy in moderate-to-severe participants. Removing severity as a confounding variable remains an important consideration in progressing knowledge in this field.

## Modeling and Simulation of Cerebral Atrophy

We reviewed the literature on spatial patterns of atrophy with a special view towards making informed recommendations to accurately simulate brain volume loss. To our knowledge, computer simulations have not yet been used to study brain atrophy in response to traumatic brain injury. We close our review with a first attempt to model and simulate brain atrophy and discuss the potential use and future application of cerebral atrophy simulation.

We propose to model cerebral atrophy as a change in tissue volume, which implies that the damaged tissue is resorbed while the density of the remaining tissue remains unchanged.^6^ We adopt our model for tissue volume changes during human brain development based on the multiplicative decomposition of the deformation gradient, **F** = ∇**φ** = **F**^e^·**F**^a^, into an elastic part **F**^e^ and an atrophy part **F**^a^.^16^ We assume that atrophy affects the tissue isotropically and represent the atrophy tensor, **F**^a^ = *υ*^1/3^**I**, through the identity tensor **I** scaled by the amount of atrophy *υ*. Values of *υ* = 1 characterize a constant brain volume, *J*^a^ = det(**F**^a^) = *υ*; values of *υ* < 1 characterize a volume loss. We can then express the elastic tensor, **F**^e^= **F**/*υ*^1/3^, as the atrophy-scaled total deformation gradient, **F **= ∇**φ**, and the elastic Jacobian, *J*^e^ = *J*/*υ*, as the atrophy-scaled total Jacobian, *J* = det(**F**). From the elastic tensor **F**^e^ and elastic Jacobian *J*^e^, we calculate the Cauchy stress **σ**(**F**^e^, *J*^e^) that enters the equilibrium equation, div(**σ**) = **0**. We represent the tissue to a first approximation as a quasi-incompressible, hyperelastic neo Hookean material with shear moduli of 2.07 and 1.15 kPa for gray and white matter, which we had previously determined from triaxial testing of human brain samples.^7^ To model *generalized* atrophy across the entire brain, we gradually atrophy the tissue at a rate of *dυ*/*dt* = − 0.4%/month in gray and *dυ*/*dt* = − 0.2%/month in white matter^63^ and simulate a 4-year period towards a total volume loss of Δ*υ* = − 20% in gray and Δ*υ* = − 10% in white matter.^44^,^52^ To model *focal* atrophy in either gray or white matter, we simulate the individual volume loss of either Δ*υ* = − 50% in gray or Δ*υ* = − 25% in white matter and compare the resulting deformation and stress profiles.

**Figure 3:**
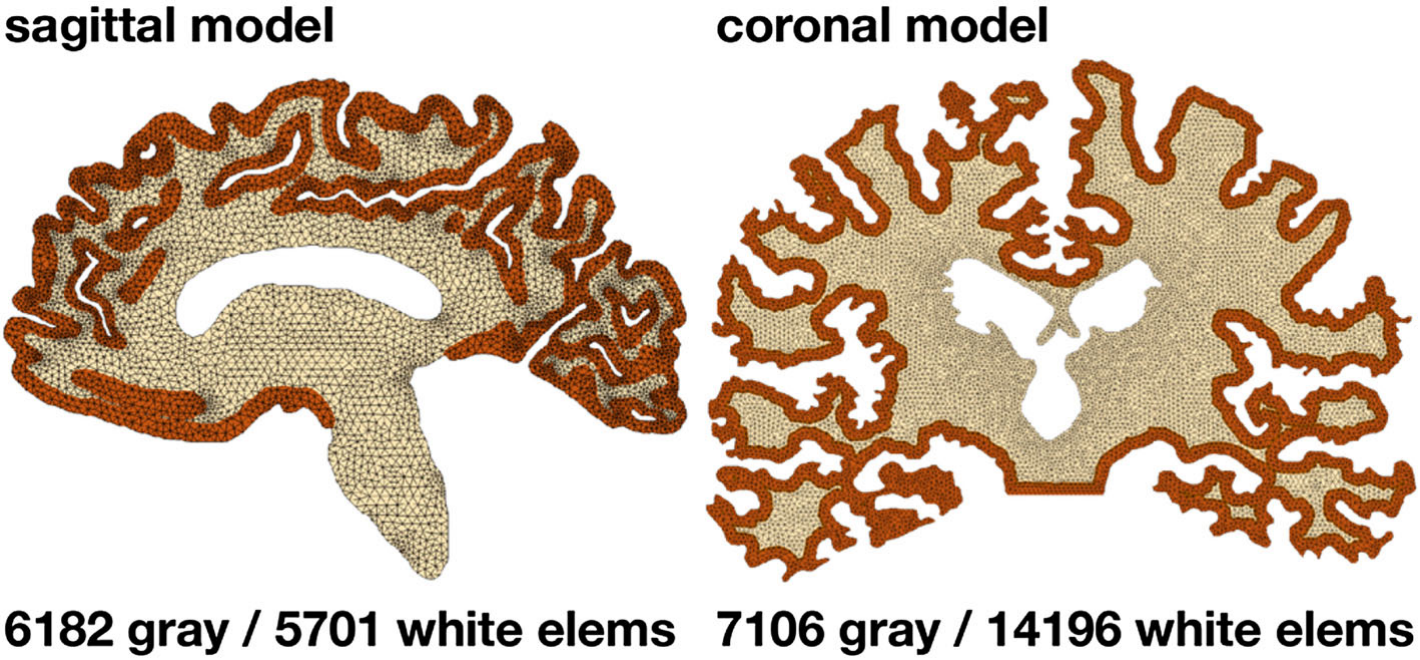
Finite element models for cerebral atrophy. Two-dimensional sagittal model with 6182 gray and 5701 white matter linear triangular elements, 6441 nodes, and 12,882 degrees of freedom, left, and coronal model with 7106 gray and 14,196 white matter linear triangular elements, 11,808 nodes, and 23,616 degrees of freedom, right, created from the magnetic resonance images in Fig. 1.

Figure 3 illustrates our two-dimensional sagittal and coronal finite element models created from magnetic resonance images. Both models distinguish between gray and white matter tissue; the sagittal model consists of 6182 gray and 5701 white matter linear triangular elements, 6441 nodes, and 12,882 degrees of freedom and the coronal model—in fact a discretization of the atrophy images in Fig. 2,^39^—consists of 7106 gray and 14,196 white matter linear triangular elements, 11,808 nodes, and 23,616 degrees of freedom. In both cases, sagittal and coronal, we apply Dirichlet boundary conditions at the brain stem and fix it in space while all other nodes are allowed to move freely upon tissue atrophy.

**Figure 4:**
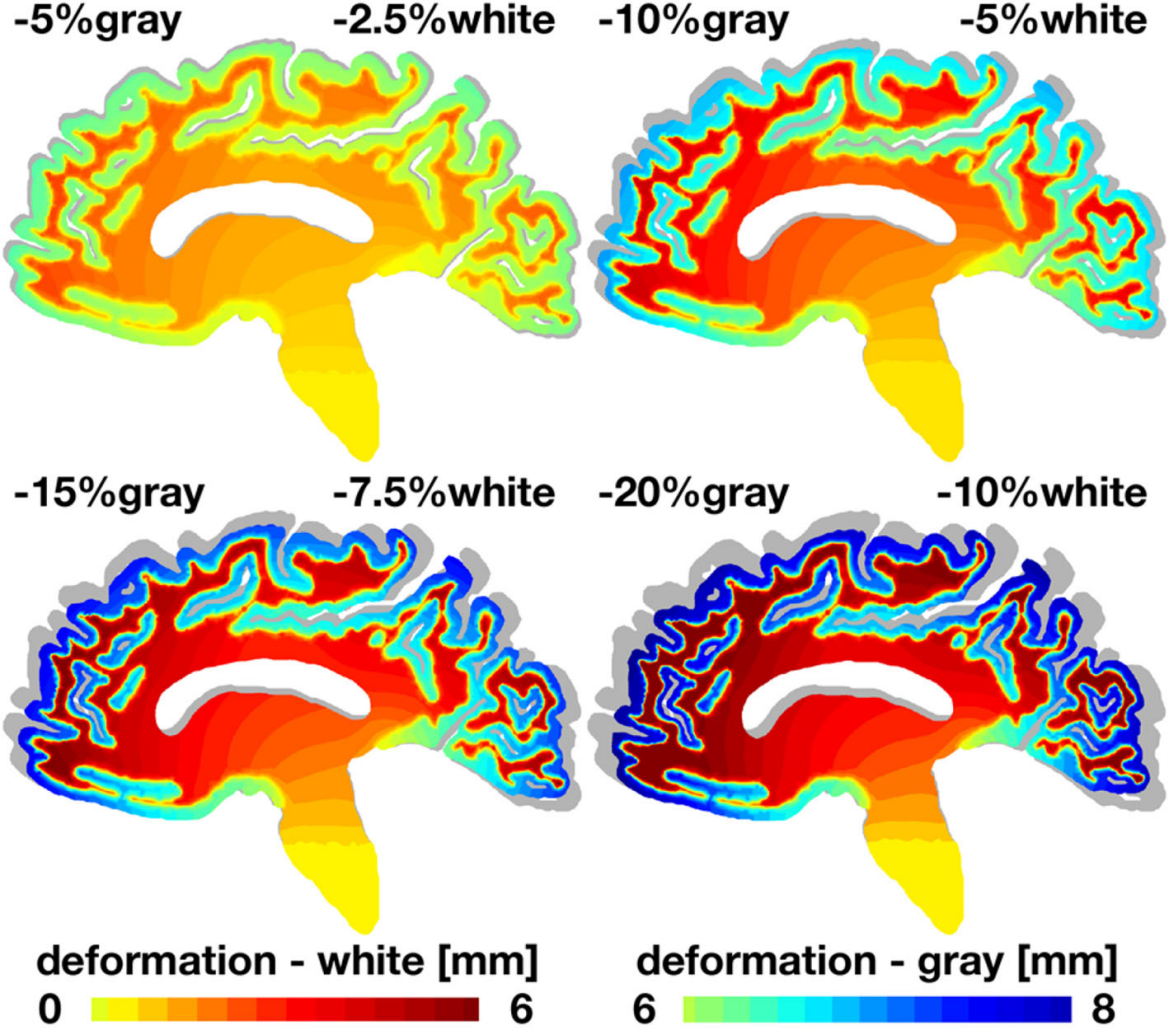
General atrophy induces sagittal gray and white matter deformation. With progressive atrophy, from − 5% in gray and − 2.5% in white matter, top left, to − 20% in gray and − 10% in white matter, bottom right, the overall deformation increases up to 8 mm. For reference, the atrophied sagittal sections are overlaid on top of the initial geometry shown in gray. In agreement with clinical pathologies, cerebral atrophy induces a marked widening of the cortical sulci, here visible through a pronounced widening of the paracentral and marginal sulci.

**Figure 5:**
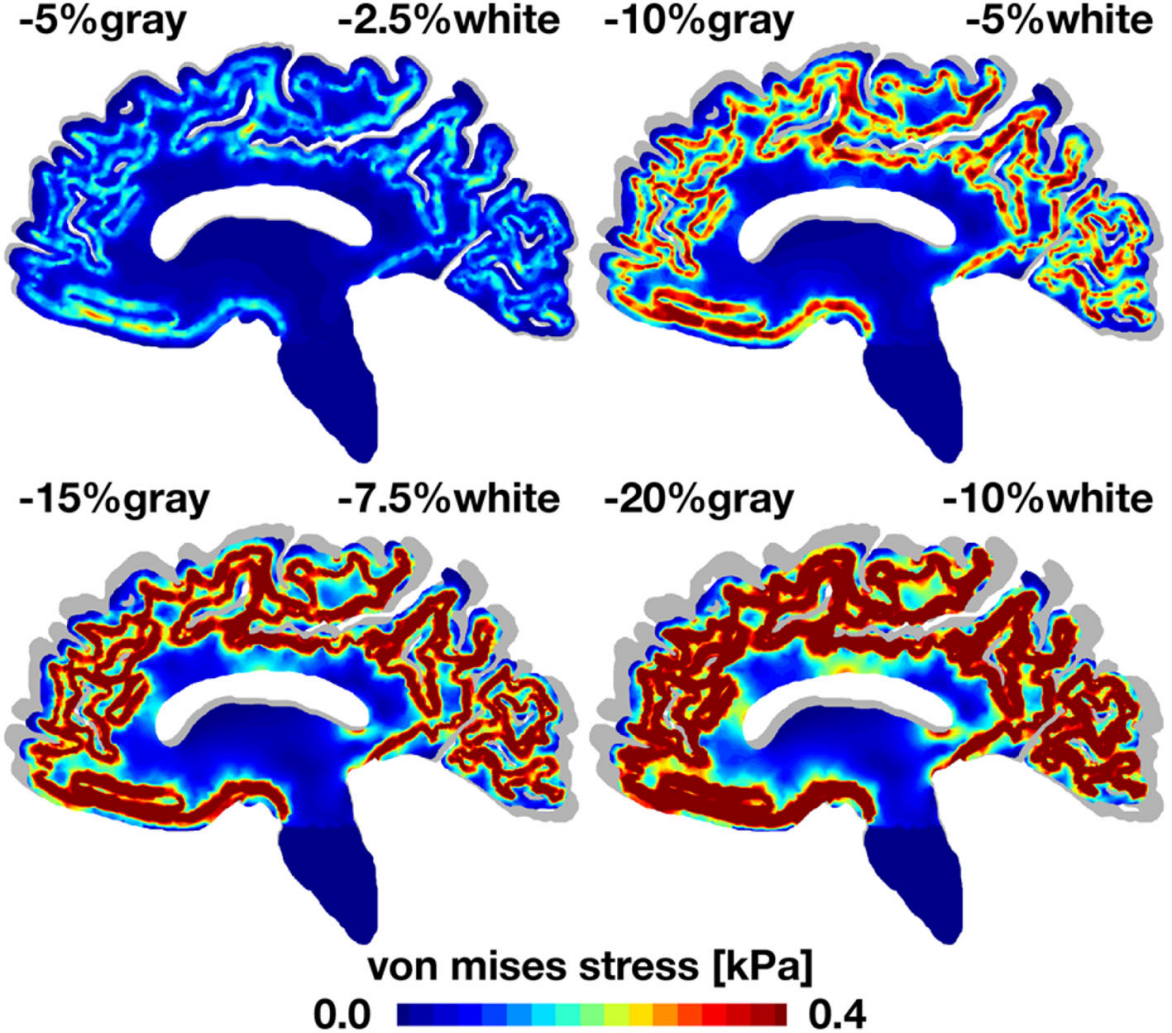
General atrophy induces sagittal shear stresses at the gray and white matter interface. With progressive atrophy, from − 5% in gray and − 2.5% in white matter, top left, to − 20% in gray and − 10% in white matter, bottom right, the von Mises stress increases up to 0.4 kPa. For reference, the atrophied sagittal sections are overlaid on top of the initial geometry shown in gray. In agreement with clinical pathologies, stress concentrations originate at the bottom of cortical sulci where lesions are concentrated.

Figures 4 and 5 illustrate the deformation and stress patterns introduced by general atrophy in the sagittal cross section. For reference, we display the atrophied sagittal sections on top of the initial sagittal geometry shown in gray. With progressive atrophy, from − 5% in gray and − 2.5% in white matter to − 20% in gray and − 10% in white matter, the overall deformation increases up to 8 mm. In agreement with clinical pathologies, cerebral atrophy induces a marked widening of the cortical sulci.^45^ Widening is most pronounced in the paracentral sulcus and the marginal sulcus. With progressive atrophy, the von Mises stress increases up to 0.4 kPa. The von Mises stress, a measure of the shear stress in the tissue, concentrates at the interface between gray and white matter. In agreement with clinical pathologies, stress concentrations originate at the bottom of the cortical sulci, at the locations where tissue histology reveals the concentration of lesions.^45^

**Figure 6:**
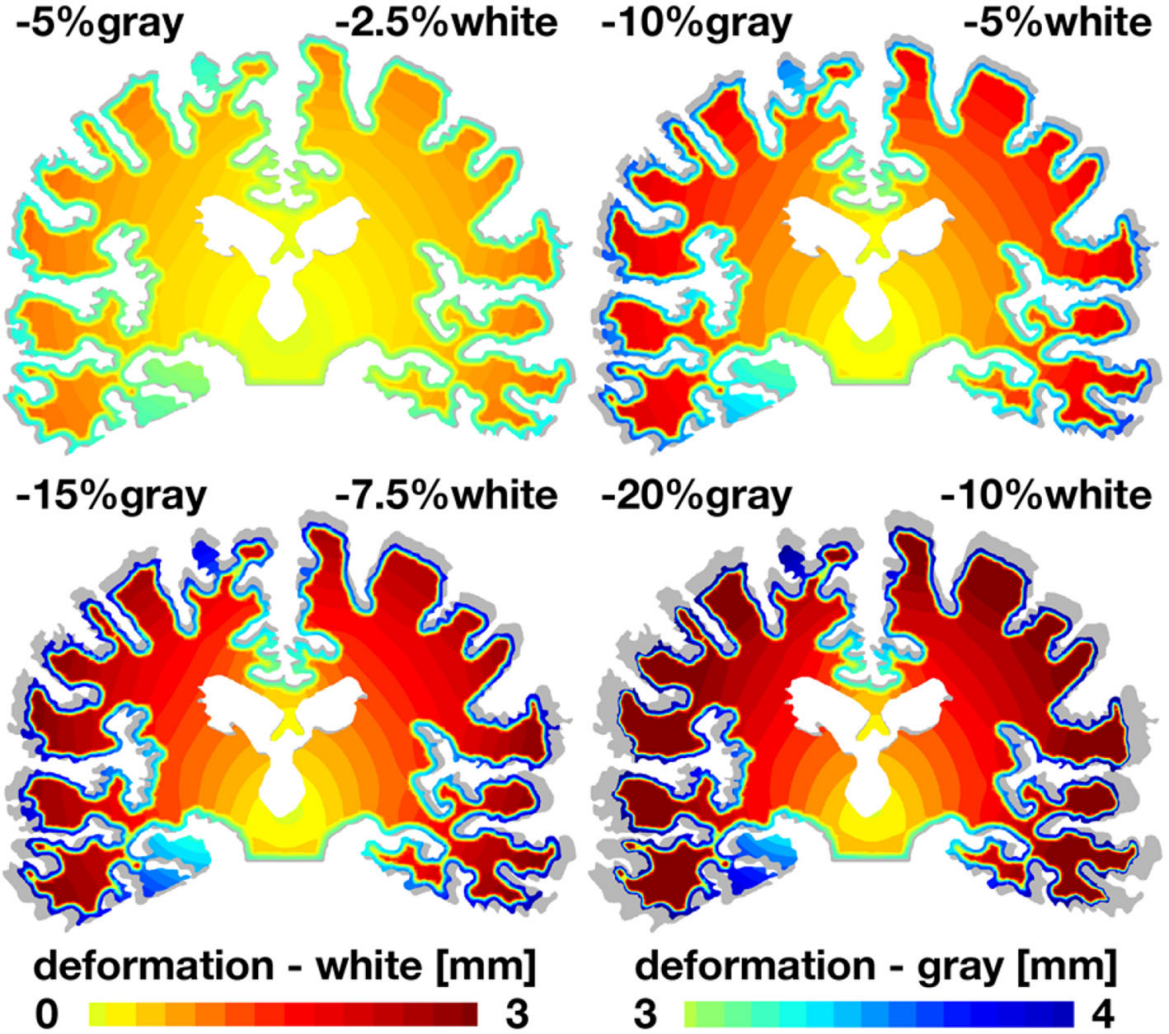
General atrophy induces coronal gray and white matter deformation. With progressive atrophy, from − 5% in gray and − 2.5% in white matter, top left, to − 20% in gray and − 10% in white matter, bottom right, the overall deformation increases up to 4 mm. For reference, the atrophied coronal sections are overlaid on top of the initial geometry shown in gray. In agreement with clinical pathologies, cerebral atrophy induces a widening of the cortical sulci, here visible through a pronounced widening of the Sylvian fissure, the superior and inferior temporal sulci, and the collateral sulcus. In agreement with the time line of atrophy in Fig. 1, the coronal model predicts a gradual enlargement of the ventricles and a notable progressive hippocampal atrophy.

**Figure 7:**
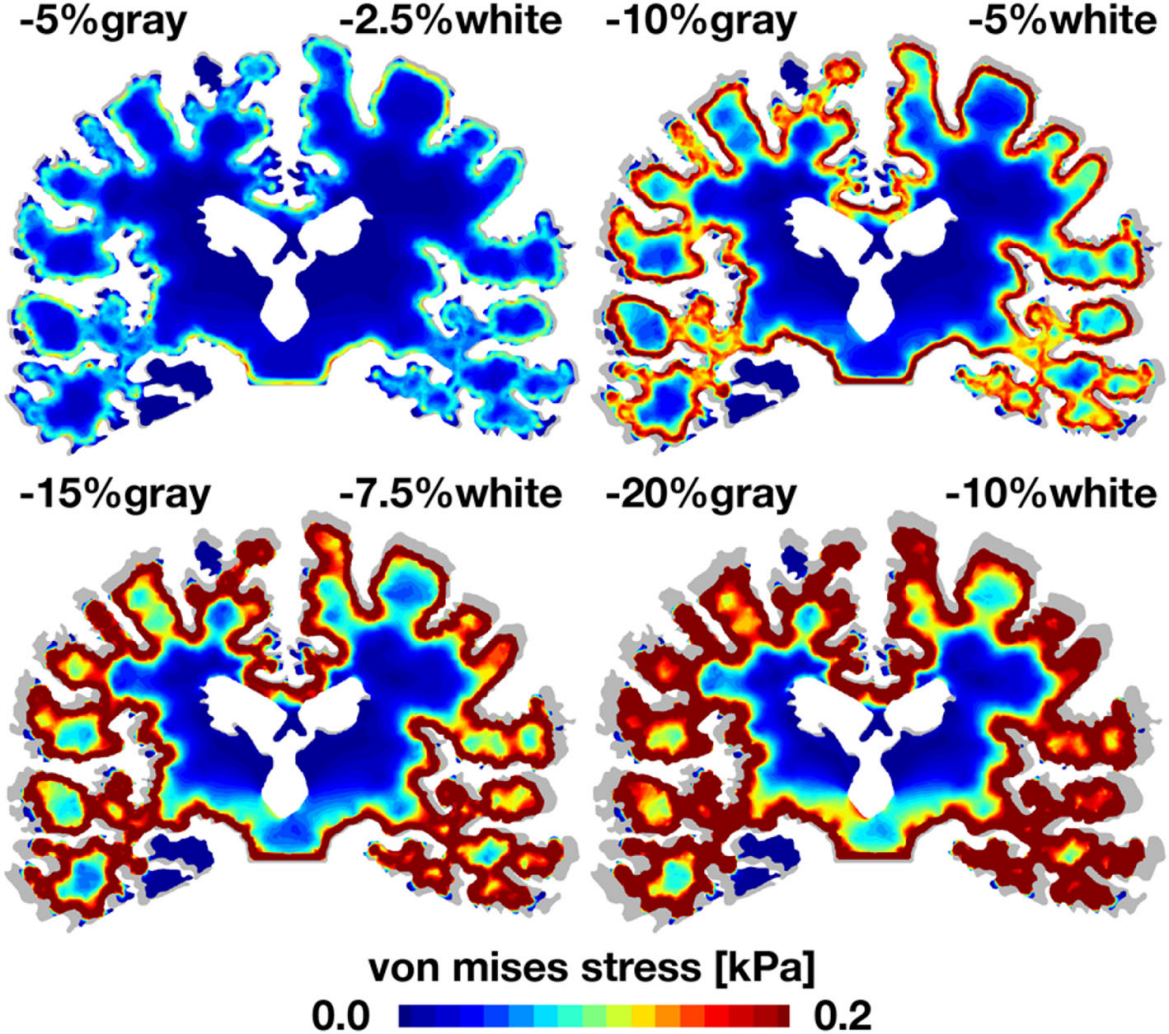
General atrophy induces coronal shear stresses at the gray and white matter interface. With progressive atrophy, from − 5% in gray and − 2.5% in white matter, top left, to − 20% in gray and − 10% in white matter, bottom right, the von Mises stress increases up to 0.2 kPa. For reference, the atrophied coronal sections are overlaid on top of the initial geometry shown in gray. In agreement with clinical pathologies, stress concentrations originate at the bottom of cortical sulci where lesions are concentrated.

Figures 6 and 7 illustrate similar deformation and stress patterns from general atrophy in the coronal cross section. As the degree of atrophy increases, from − 5% in gray and − 2.5% in white matter to − 20% in gray and − 10% in white matter, the overall deformation increases up to 4 mm. Similar to the sagittal model, the coronal model reveals a marked widening of the cortical sulci in agreement with clinical observations. In the coronal model, we clearly observe a pronounced widening of the Sylvian fissure, the superior and inferior temporal sulci, and the collateral sulcus. In agreement with the clinical timeline of atrophy in Fig. 2, the coronal model predicts a gradual enlargement of the ventricles and a notable progressive hippocampal atrophy.^10^ As atrophy progresses, the von Mises stress increases up to 0.2 kPa, again with largest stress mainly at the interface between gray and white matter. Similar to the sagittal model, the coronal model shows pronounced stress concentrations at the bottom of the cortical sulci, most visible in the top left image. This observation agrees well with histological findings, where lesions are first observed at the bottom of the cortical sulci.

**Figure 8:**
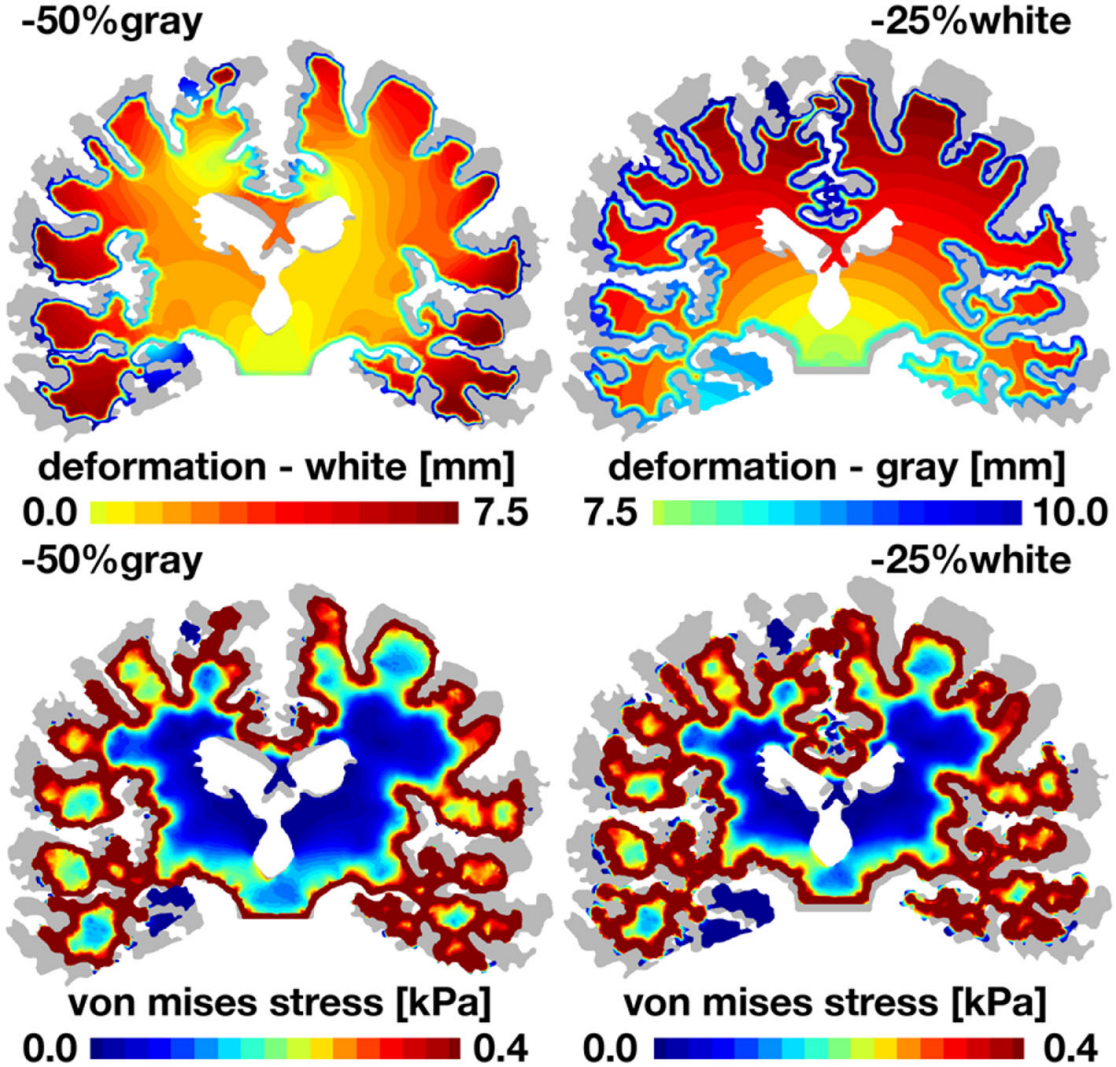
Focal gray and white matter atrophy induces coronal gray and white matter deformation and shear stresses. Pure gray matter atrophy, left, and pure white matter atrophy, right, generate markedly different deformation profiles, top, and stress profiles, bottom. Focal gray matter atrophy induces cortical thinning, a widening of the cortical sulci, and an enlargement of the ventricles. Focal matter atrophy induces a contraction of the whole brain toward the brain stem. For reference, the atrophied coronal sections are overlaid on top of the initial geometry shown in gray.

Figure 8 highlights the regional differences between focal gray and white matter atrophy. Our simulations reveal that pure gray matter atrophy and pure white matter atrophy generate markedly different deformation and stress profiles although the underlying deformations and stresses are on the same order of magnitude, up to 10 mm and 0.4 kPa. Focal gray matter atrophy induces a widening of the cortical sulci, an enlargement of the ventricles, and a pronounced cortical thinning.^45^ In contrast, focal matter atrophy induces a contraction of the whole brain toward the brain stem while the cortical thickness remains unchanged. Based on these first prototype simulations and our current knowledge of injury-induced brain tissue atrophy, we make the following recommendations for creating atrophy simulation tools.

First, simulations should aim to account for injury severity. Our review reveals that mild injury pathology deviates greatly from the pathology of moderate-to-mild injury. Not only do the spatial patterns of atrophy differ amongst the two groups, but the literature suggests that severity also impacts magnitude of volume loss. For the mild traumatic brain injury population, it may be pertinent to combine the conclusions in the literature to simulate atrophy by a − 4.16% change of brain parenchyma volume^43^ in the pericalcarine region of the gray matter.^53^ These numbers roughly correspond to the top left images of our simulations with − 5% gray and − 2.5% white matter atrophy in Figs. 4, 5, 6 and 7. We acknowledge that the current knowledge of spatial patterns of atrophy in mild traumatic brain injury patients is sparse, and, thus, such suggestions may be debatable at best. However, suggestions regarding the moderate-to-severe traumatic brain injury population may be more illuminating, given the comparatively greater amount of information about this group.

Second, here we have simulated generalized atrophy across the whole brain and focal atrophy in gray and white matter. For example, for a focal gray matter atrophy of − 50% in Fig. 8, our model predicts a cortical thinning of − 21%, which agrees well with the observations in the literature varying regionally between − 10.7% and − 20.4%.^59^ For future simulations, we propose to simulate focal atrophy in selected regions of the gray matter including the bilateral thalamus, bilateral hippocampus, cerebellum, bilateral putamen, bilateral pallidum, and insula, and regions of the white matter including the corpus callosum, corona radiata, capsule, brainstem, and inferior and superior fascicules. Ideally, we would correlate these regions of pronounced atrophy to damage regions within the brain, either inferred from a single impact or from repeated subconcussive heads^32^ in accordance with a damage nucleation model.^31^ Given that these areas have the greatest consensus of significant tissue loss in the literature, it would be interesting to explore how their regional atrophy changes the deformation and stress profiles of the surrounding brain structures. Since brain injuries have historically been associated with elevated shear, the von Mises stress highlighted in Figs. 5, 7, and 8 seems to be a reasonable proxy for tissue damage.^17^ Based on the fact that there is quantitative data on volume loss of some but not all of these structures, we recommend using the estimates of overall volume loss and evenly spread the loss across all aforementioned structures. For example, investigators have reported a brain loss between − 4.0% and − 8.5% within 1 year of injury.^44^,^52^ We therefore recommend to atrophy each structure equally such that overall percent tissue loss ranges between − 4% and − 8.5%. Given the correlation between severity and atrophy, worse injuries should result in greater overall percent loss, closer to − 8.5%, whereas simulations of more moderate injury should use the lower estimate, closer to − 4%. These numbers roughly correspond to the top left and top right images of our simulations with − 5% and − 10% of gray matter atrophy in Figs. 4, 5, 6 and 7.

Third, although it would add another level of complexity to the atrophy simulation, age, and probably even gender, should be considered since both are clearly correlated to the initial brain dimensions and the degree of atrophy, even in the healthy population. As ages increase so should percent tissue loss. Long-term chronic studies should correct for these effects.

Finally, as for all simulations, it would be interesting to expand our two-dimensional prototype simulations into three dimensions, specifically with a view towards more accurate boundary conditions. In addition, to more thoroughly investigate stress profiles in response to tissue atrophy, it is paramount to accurately represent the *in vivo* constitutive behavior of the living brain,^67^ particularly at the gray and white matter interface, where Figs. 5, 7, and 8 identify regions of local stress concentrations. While locations of these stress concentrations are likely relatively robust with respect to stiffness changes, the absolute stress values will strongly depend on the brain stiffness, which could change significantly in response to injury. Along the same lines, it is important to more tightly correlate tissue elasticity to tissue microstructure.^65^ This could also imply replacing the purely isotropic atrophy tensor, **F**^a^ = *υ*^1/3^**I**, by a more microstructurally-based atrophy tensor, **F**^a^ = *υ*^iso^**I **+*υ*^ani^**n ⊗ n**, that explicitly accounts for tissue loss along pronounced axonal directions **n**.^28^ Last, and most importantly, as a first step, we have only simulated generalized cerebral atrophy in Figs. 4, 5, 6 and 7 and focal atrophy of gray and white matter in Fig. 8. We have quantitatively prescribed the gray and white matter volume loss, i.e., the amount of atrophy Δ*υ* and the atrophy rate *dυ*/*dt* from clinically reported values and qualitatively compared the resulting atrophy patterns against clinical pathologies. The logical next step would be to model atrophy as a result of biochemical or biomechanical events.^66^ Ideally, this would imply applying acceleration profiles recorded during the event of an injury, identifying regions in which tissue strains, strain rates, or stresses exceed a critical safety level threshold,^37^ and exploring the effects of the resulting personalized atrophy profiles on structure and function.

## Conclusion

We believe this review has important implications for the accurate modeling and simulation of brain atrophy, which can be used in both a clinical and research setting. We recognize the potential of chronic atrophy simulations in predicting long-term injury outcomes. Beyond the structural changes associated with cerebral atrophy, gray and white matter degeneration clearly correlates with region-specific functional changes. This suggests that longitudinal atrophy analyses from magnetic resonance images could potentially be used as a diagnostic tool to quantify the onset and progression of neurodegeneration in response to multiple repeated subconcussive impacts. Understanding a patient’s projected atrophy pattern may provide physicians the opportunity to better prepare patients and their families for possible injury outcomes including dementia, aphasias, or even behavioral and mood alterations. With growing research on possible interventions in the acute phases of traumatic brain injury, physicians could potentially utilize predictive atrophy simulations to decide for whom, how, and when to administer pharmaceutical treatment.

From a research perspective, brain atrophy simulations are imperative to reveal the interplay between acute biomechanical factors and chronic biochemical events in neurodegeneration. Increasing evidence suggests that elevated mechanical shear in the brain parenchyma triggers a neurodegenerative cascade. Understanding the strains and stresses across the brain, before, during, and after neurodegeneration will illuminate whether the atrophy itself introduces additional destructive mechanical factors. While it is immediately intuitive that an increase in brain volume during swelling or tumor growth induces an elevated pressure and thereby directly affects the mechano-biochemical environment within the skull, the same could be true for a decrease in volume during neurodegeneration that induces a shift in tissue homeostasis. It could very well be that atrophy-induced deformations and stresses locally exceed the tissue tolerance and trigger a negative feedback loop in which more atrophy causes further deformation. Probing to which extent cerebral atrophy triggers neurodegeneration using computer simulations could provide an exciting and new direction for future research.
